# The Incidence of Postoperative Shoulder Stiffness After Arthroscopic Rotator Cuff Repair: A Systematic Review

**DOI:** 10.7759/cureus.37199

**Published:** 2023-04-06

**Authors:** Anthony N Baumann, Caleb Oleson, Deven P Curtis, Thad Indermuhle, John Martin Leland

**Affiliations:** 1 Department of Rehabilitation Services, University Hospitals, Cleveland, USA; 2 College of Medicine, Northeast Ohio Medical University, Rootstown, USA; 3 Department of Orthopedic Surgery, University Hospitals, Cleveland, USA

**Keywords:** postoperative, rotator cuff repair, shoulder, arthroscopic, shoulder stiffness

## Abstract

Rotator cuff tear (RCT) is a common shoulder condition frequently treated with arthroscopic rotator cuff repair (ARCR) after conservative interventions have failed. Postoperative shoulder stiffness (POSS) is a common complication after ARCR. The purpose of this study is to determine the incidence of POSS after ARCR in total and based on tear extent prior to ARCR. A systematic review was performed using PubMed, Cumulative Index of Nursing and Allied Health Literature (CINAHL), Medical Literature Analysis and Retrieval System Online (MEDLINE), and ScienceDirect databases. The search terms were “rotator cuff repair” AND “arthrofibrosis” OR “postoperative shoulder stiffness.” Articles were selected based on ARCR and the presence of POSS documentation. Twenty-five articles out of 284 articles met the final selection criteria after reviewing for patients who received ARCR. Out of all patients (n=9,373), 597 had POSS (6.4%). Out of the 2,424 patients with a specified tear pattern, 96 out of 1,862 (5.2%) patients with full-thickness tears and 58 out of 562 (10.3%) patients with partial-thickness tears had POSS after ARCR. Partial-thickness tears are associated with higher rates of POSS after ARCR as compared to full-thickness tears (p<0.001). Overall, POSS is a common complication after ARCR with an incidence of 6.4%, regardless of tear type. POSS is more common in patients who receive ARCR for partial-thickness RCT (10.3%) as compared to patients who receive ARCR for full-thickness RCT (5.2%). More research is needed to determine other factors impacting the incidence of POSS after ARCR.

## Introduction and background

Arthroscopic rotator cuff repair (ARCR) is a common surgical intervention viewed as the gold standard to address a rotator cuff tear (RCT) for which nonoperative treatment has failed [1-3]. ARCR is one of the most common surgeries performed by orthopedic surgeons with increasing frequency in recent years [2]. While ARCR can have various postoperative complications, postoperative shoulder stiffness (POSS) is a postoperative complication that is associated with increased cost and decreased patient outcomes [4,5]. Furthermore, POSS has been reported as a common complication after ARCR in the literature [1,2]. The exact prevalence of POSS is unknown, with estimated incidence rates varying from 2% to 28% [1,6]. Furthermore, the incidence of POSS after ARCR has been shown to increase in the presence of risk factors, such as diabetes [7]. Other risk factors for POSS after ARCR, such as preoperative shoulder stiffness, female sex, operative technique, partial supraspinatus tear, and prolonged immobilization, have been reported elsewhere in the literature [6,7]. Despite these numerous potential risk factors for POSS after ARCR, predicting POSS continues to remain a clinical challenge [7]. Although large database studies have been used to assess the rate of other complications after ARCR, POSS is a clinical complication that is not easily assessed with database data extraction studies, adding to the lack of precision on the incidence of POSS as well as the prediction of POSS after ARCR [2].

POSS after ARCR is a relevant concern as the development of POSS after ARCR has been reported to increase patient dissatisfaction and may decrease functional outcomes [6,8]. Furthermore, POSS after an otherwise successful ARCR can damage the relationship between the orthopedic surgeon and the patient due to patient distress and dissatisfaction [6,7]. One previous systematic review on the incidence of POSS after ARCR is over a decade old with a relatively small sample size of six articles and 1,064 patients [9]. With the advent of more literature on POSS after ARCR in recent years, a new systematic review is warranted to better assess the incidence of POSS after ARCR in total and by tear type. Increased understanding of the risk of POSS after ARCR may be used to prevent negative outcomes associated with POSS, especially as the number of ARCR surgeries per year is increasing [2]. To our knowledge, no other systematic review has been performed that examined the incidence of POSS after ARCR based on partial-thickness or full-thickness RCT. The purpose of the current study is to determine the incidence of POSS after ARCR for partial-thickness and full-thickness rotator cuff tears to help orthopedic surgeons and physical therapists improve patient outcomes and reduce complications.

## Review

Methods

The current study is a systematic review of all relevant and full-text articles regarding POSS after ARCR found on the PubMed, Cumulative Index of Nursing and Allied Health Literature (CINAHL), Medical Literature Analysis and Retrieval System Online (MEDLINE), and ScienceDirect databases without time restrictions. The current study included articles published up until the time of October 21, 2022, which was when the databases were searched with the full search terms. The full search terms used to retrieve articles in the four databases were “rotator cuff repair” AND “arthrofibrosis” OR “postoperative shoulder stiffness.” Multiple authors helped with article selection as well as data extraction. The article types included in the review were randomized controlled trials, retrospective cohort studies, and prospective cohort studies. Articles were initially screened by title and abstract. Articles were included if any number of cases of POSS, including zero cases, were specifically reported. For the current study, POSS was defined as any type of POSS, including postoperative arthrofibrosis and postoperative adhesive capsulitis. Studies were excluded from the final review if they did not report POSS incidence in the study population, did not report postoperative complications, or did not perform ARCR for rotator cuff repair. Furthermore, systematic reviews, meta-analyses, case series, and case reports were excluded from the study. Articles were selected based on full text and relevance based on ARCR and the occurrence of POSS of any type or severity. Articles were also grouped into categories by the type of RCT surgically corrected by ARCR. Groups were created by the information listed in the individual articles and included “partial” for partial-thickness tears, “full thickness” for full-thickness tears, and “all tears” if the tear type was not specified or used in the subgroup analysis. The chi-square test was used for statistical analysis to compare the incidence of ARCR for partial-thickness tears to the incidence of ARCR for full-thickness tears.

Results

**Figure 1:**
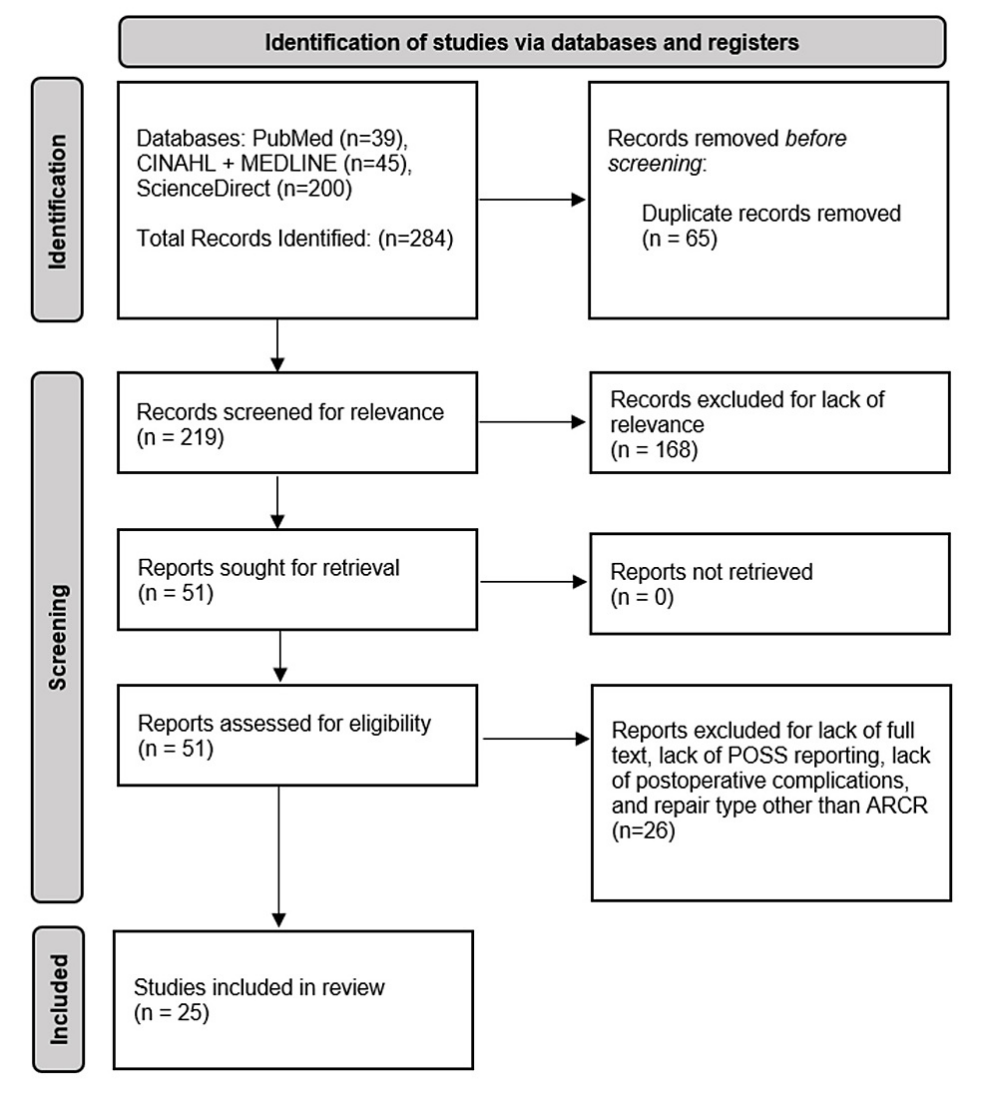
2020 PRISMA flow diagram model describing the records identified, articles screened by abstract and title, and number of included full-text articles after the final selection criteria were applied for the current systematic review PRISMA: Preferred Reporting Items for Systematic Reviews and Meta-Analyses, POSS: postoperative shoulder stiffness, ARCR: arthroscopic rotator cuff repair

A total of 284 articles were retrieved from PubMed, CINAHL, MEDLINE, and ScienceDirect with 25 articles meeting the final selection criteria for inclusion in the systematic review [10-29]. Figure 1 shows the Preferred Reporting Items for Systematic Reviews and Meta-Analyses (PRISMA) flow diagram for the identification, screening, and final selection of the included articles.

Of the 25 articles selected for the study, two studies were Level I evidence, three studies were Level II evidence, five studies were Level III evidence, and 15 studies were Level IV evidence. Table 1 shows more details on the 25 articles selected for the current systematic review [10-29]. The 25 articles selected for final inclusion had a total of 9,373 patients who underwent ARCR for RCT, regardless of tear type. The total incidence of POSS after ARCR was 6.4%, with 597 out of 9,373 patients reporting POSS after surgery. Out of the 9,373 patients who underwent ARCR, 2,424 patients were included in articles that specified the tear type as either partial-thickness or full-thickness RCT. Out of the 2,424 patients with specified tear type listed in the article, 96 out of 1,862 (5.2%) patients who underwent ARCR for full-thickness RCT had POSS as compared to 58 out of 562 (10.3%) patients who underwent ARCR for partial-thickness RCT. Table 2 shows the incidence of POSS after ARCR based on tear type from the selected articles. Overall, patients who underwent ARCR for partial-thickness RCT had a significantly higher incidence of POSS as compared to patients who underwent ARCR for full-thickness RCT (p<0.01).

**Table 1:** Description of studies with a reported incidence of POSS after ARCR Information includes first author, year of publication, level of evidence, type of study, type of tear, number of patients, number of cases of POSS, and incidence of POSS in each study (percentage). POSS: postoperative shoulder stiffness, ARCR: arthroscopic rotator cuff repair

Number	Author (year)	Level of evidence	Type of study	Type of tear	Number of patients	Cases of POSS	Incidence of POSS
1	Shin (2018) [2]	II	Randomized controlled trial	Partial	48	5	10.4%
2	Smolen (2020) [10]	IV	Prospective cohort	All tears	50	6	12%
3	Cole (2007) [11]	IV	Prospective cohort	Full thickness	47	0	0%
4	Ranalletta (2016) [12]	IV	Prospective cohort	Partial	80	5	6.3%
5	Youm (2005) [13]	IV	Retrospective cohort	All tears	42	2	4.8%
6	Felsch (2021) [14]	IV	Retrospective cohort	Partial	350	39	11.1%
	Felsch (2021) [14]	IV	Retrospective cohort	Full thickness	1,311	88	6.7%
7	Teratani (2019) [15]	III	Retrospective cohort	All tears	213	17	8%
8	Toussaint (2011) [16]	IV	Retrospective cohort	All tears	154	17	11%
9	Sheps (2019) [17]	I	Randomized controlled trial	Full thickness	206	3	1.5%
10	Guity (2021) [18]	IV	Retrospective cohort	All tears	335	121	36.1%
11	Miyazaki (2009) [19]	IV	Retrospective cohort	All tears	61	2	3.3%
12	Vap (2017) [20]	IV	Retrospective cohort	Partial	20	0	0%
13	Tan (2020) [21]	IV	Retrospective cohort	All tears	290	32	11%
14	Cho (2022) [6]	IV	Retrospective cohort	All tears	274	39	14.2%
15	Huberty (2009) [8]	IV	Retrospective cohort	All tears	489	24	4.9%
16	Yeazell (2022) [22]	III	Prospective cohort	Partial	64	9	14.1%
17	Wang (2022) [23]	III	Retrospective cohort	All tears	3,266	36	1.1%
18	Posada (2000) [24]	IV	Prospective cohort	Full thickness	60	2	3.3%
19	Jenssen (2018) [25]	I	Prospective cohort	Full thickness	118	2	1.7%
20	Audigé (2021) [4]	III	Retrospective cohort	All tears	1,330	112	8.4%
21	Schneider (2021) [26]	III	Retrospective cohort	All tears	126	9	7.1%
22	Blonna (2017) [7]	II	Prospective cohort	All tears	31	7	22.6%
23	Shin (2012) [27]	II	Prospective control	Full thickness	120	1	0.8%
24	Cucchi (2020) [28]	IV	Prospective cohort	All tears	237	19	8%
25	Takahashi (2022) [29]	IV	Retrospective cohort	All tears	51	0	0%

**Table 2:** Incidence of POSS after ARCR in total and based on tear type POSS: postoperative shoulder stiffness, ARCR: arthroscopic rotator cuff repair

Category	Number of patients	Cases of POSS	Incidence of POSS
Total ARCR	9,373	597	6.4%
Tear type specified	2,424		
Full thickness	1,862	96	5.2%
Partial thickness	562	58	10.3%

Discussion

The current systematic review provides an updated incidence of POSS after ARCR based on tear type with one of the largest number of articles and patients to date on the topic of POSS. Knowledge concerning POSS after ARCR is crucial as POSS has been associated with decreased patient satisfaction after surgery, increased cost, and decreased outcomes [1,4-6]. Therefore, the ability to better understand POSS after ARCR is paramount to ideal patient outcomes [6]. The current study found that the overall incidence of POSS after ARCR, regardless of tear type, was 6.4% in a population of 9,373 patients.

In the literature, the previously reported incidence levels of POSS after ARCR have been highly variable with rates reported as high as 35% three months after ARCR [6]. The current systematic review did not assess the incidence of POSS after ARCR at different postoperative timelines but rather examined the incidence of POSS at any point after ARCR. Therefore, the results from the current study indicate that the true incidence of POSS at any time after ARCR is much closer to the lower end of the reported incidence of POSS in the literature. It is important to note that POSS after ARCR can range in severity with some cases resistant to nonoperative treatment [9]. One systematic review categorized POSS in their systematic review into either “transient” POSS or “resistant” POSS based on positive or negative responses to nonoperative treatment, respectively [9]. In that systematic review, the incidence of transient POSS was 10% and the incidence of resistant POSS was 3.3% [9]. The management of POSS after ARCR usually involves conservative treatment options such as oral nonsteroidal anti-inflammatory drugs and physical therapy [5]. A small number of cases of POSS after ARCR require arthroscopic capsular release [5]. It is possible that POSS after ARCR might be able to be predicted in patients with risk factors, which could help decrease further complications, additional procedures, and diminished outcomes [7]. Diabetes mellitus and increased time until rehabilitation after ARCR have been reported as independent risk factors for POSS in the literature [6]. Recently, the relationship between rehabilitation usage after ARCR and tear type has been investigated with ARCR for partial-thickness rotator cuff tears being associated with increased postoperative physical therapy [30]. One possible reason for this association could be the increased incidence of POSS in patients with ARCR for partial-thickness tears as indicated by the current study.

The current study found a significantly larger incidence of POSS after ARCR for partial-thickness tears at 10.3% as compared to an incidence of 5.2% of POSS after ARCR for full-thickness tears. This finding of increased incidence of POSS in patients after ARCR for partial-thickness tears has been reported elsewhere in the literature [4]. Interestingly, POSS has some positive attributes as POSS has been shown to have a protective effect for re-tear after ARCR [31]. The protective effect of POSS against re-tear can be beneficial; however, POSS can linger for years, likely contributing to decreased patient satisfaction [5].

One limitation of the current study is that the severity of POSS and the subsequent management, whether operative or nonoperative, is not known. Another limitation of this study is that the incidence of POSS after ARCR based on patient age or different time points after surgery is unknown. More research is needed to determine the incidence of POSS with a larger sample size that requires conservative versus surgical intervention and factors in patient age and follow-up time.

Another limitation that complicates the discussion of POSS after ARCR is the lack of consistency in the definition of POSS. Many articles included in the current study did not provide definitions of POSS. Others provided various definitions of POSS after ARCR, which is consistent with the literature that indicates a wide variability in POSS definition [6]. Understanding that some studies may rely more on patient subjective complaints while others use objective range-of-motion measurements to diagnose POSS may explain the large variability in incidence previously reported in the literature. Furthermore, another study limitation of the current systematic review is the relatively high number of low-level evidence studies, potentially obstructing the true incidence of POSS after ARCR. Overall, more research is needed with higher quality studies to help identify the true incidence of POSS after ARCR to help improve patient outcomes.

## Conclusions

POSS is a common complication after ARCR with an overall incidence of 6.4%, regardless of tear type. POSS was more common in patients who underwent ARCR for partial RCTs as compared to patients who underwent ARCR for full-thickness RCTs. The incidence of POSS after ARCR for partial-thickness tears was 10.3%, while the incidence of POSS after ARCR for full-thickness tears was 5.2%. Further research is needed to determine more factors contributing to POSS after ARCR, the severity of POSS, and the management of POSS after ARCR dependent on tear type.
